# Protection of Trigonelline on Experimental Diabetic Peripheral Neuropathy

**DOI:** 10.1155/2012/164219

**Published:** 2012-12-06

**Authors:** Ji-Yin Zhou, Shi-Wen Zhou

**Affiliations:** Base for Drug Clinical Trial, Xinqiao Hospital, Third Military Medical University, Chongqing 400037, China

## Abstract

The mechanisms leading to diabetic peripheral neuropathy are complex and there is no effective drug to treat it. As an active component of several traditional Chinese medicines, trigonelline has beneficial effects on diabetes with hyperlipidemia. The protective effects and the mechanism of trigonelline on diabetic peripheral neuropathy were evaluated in streptozotocin- and high-carbohydrate/high-fat diet-induced diabetic rats. Rats were divided into four groups at the end of week 2: control, diabetes, diabetes + trigonelline (40 mg/kg), and diabetes + sitagliptin (4 mg/kg). After 48-week treatment, technologies of nerve conduction, cold and hot immersion test, transmission electron microscopy, real-time PCR, and Western blotting were applied. Serum glucose, serum insulin, insulin sensitivity index, lipid parameters, body weight, sciatic nerve conduction velocity, nociception, glucagon-like peptide-1 receptor mRNA and protein, total and phosphorylated p38 mitogen-activated protein kinases protein expression, malonaldehyde content, and superoxide dismutase activity were altered in diabetic rats, and were near control levels treated with trigonelline. Slight micropathological changes existed in sciatic nerve of trigonelline-treated diabetic rats. These findings suggest that trigonelline has beneficial effects for diabetic peripheral neuropathy through glucagon-like peptide-1 receptor/p38 mitogen-activated protein kinases signaling pathway, nerve conduction velocity, antioxidant enzyme activity, improving micropathological changes of sciatic nerve and decreasing lipid peroxidation.

## 1. Introduction

As a common complication of diabetes, diabetic peripheral neuropathy (DPN) affects likely up to one-third of adults with diabetes [1]. The most common form of DPN is a sensory polyneuropathy with symptoms such as paresthesia, unremitting pain (hyperalgesia), and reduced temperature and vibration perception thresholds. Clinical trials have failed to demonstrate the effectiveness of any drug treatment in stabilizing or improving nerve function, and only symptomatic pain therapies with variable efficacy are available [2]. First and foremost, treatment of DPN centers on control of the patient's blood glucose level. Nowadays, the only agents labeled for the treatment of DPN include lidocaine patches 5%, duloxetine, gabapentin, and pregabalin. Despite clinical use of these agents, the successful therapy of DPN remains a challenge [3]. Since pathogenic complexity of DPN, new therapeutic interventions that target primary mechanisms contributing to nerve damage are critical for the future treatment of this complication.

Recently, diabetic healthcare professions had a considerable interest in regarding complementary and alternative approaches, including by identifying natural neuroprotective constituent from herb medicine to replace synthetic ones [4, 5]. As we recently reviewed [6], trigonelline, a major ingredient of several traditional Chinese medicine with anti-diabetic effect [7, 8], has a hypoglycemic effect both in rats [9–12] and humans [13, 14] and has antioxidant effectiveness *in vitro* [15, 16]. Recently the study in our laboratory showed that trigonelline had a beneficial effect for diabetes through decreasing blood glucose and lipid levels, increasing insulin sensitivity index and insulin content, upregulating antioxidant enzyme activity, and decreasing lipid peroxidation [17]. Trigonelline also is one of the main components of *Mirabilis jalapa* L. root which possesses both potential insulin sensitivity and hypoglycemic and hypolipidemic effects on diabetes [18].

Endogenous glucagon-like peptide-1 (GLP-1) is an insulinotropic peptide synthesized and secreted from the L-cells of the gastrointestinal tract in response to food intake. When given exogenously, GLP-1 improves glucostasis in type 2 diabetic patients, primarily by stimulating endogenous insulin secretion. GLP-1 and related peptides possess neural functions including neurotrophic and neuroprotective effects, in addition to glucoregulatory and energy balance functions [19]. Dipeptidyl peptidase IV inhibitor inhibits degradation of GLP-1 by the dipeptidyl peptidase IV enzyme. Several researches show that GLP-1 and dipeptidyl peptidase IV inhibitor, such as Exendin-4 and vildagliptin, offer protection against the experimental DPN [20–24]. GLP-1 receptor stimulation preserves primary cortical and dopaminergic neurons in cellular and rodent models of stroke and Parkinsonism [25]. Peripheral nerve of diabetic rodents had functional GLP-1R and GLP-1R-mediated ERK-signaling in sciatic nerve of diabetic rodents protecting large motor fibre function and small C fibre structure by a mechanism independent of glycaemic control [26]. Diabetes is known to activate p38 MAPK in nerve tissues in both rats [27] and patients and inhibition of p38 MAPK has beneficial effects related to nerve function [28]. But the function of GLP-1R-mediated p38 mitogen-activated protein kinases (MAPK) signaling pathway in the DPN is still unknown.

With the above background, the present study systematically investigated the beneficial effect of trigonelline on peripheral neuropathy in low-dose streptozotocin and high-carbohydrate/high-fat diet-induced diabetic rats, by technologies of nerve conduction, cold and hot immersion test, transmission electron microscopy, real-time PCR, and Western blotting. The efficacy was compared with a standard hypoglycemic drug, dipeptidyl peptidase IV inhibitor, and sitagliptin.

## 2. Materials and Methods

### 2.1. Experimental Animals

Male Wistar rats, weighing 180–220 g, were purchased from Research Institute of Surgery Experimental Animal Center and bred in specific pathogen-free condition. All experiments were performed with the approval of the Animal Studies Ethics Committee of Xinqiao Hospital, Third Military Medical University. All animals were separately housed in cages (one rat per cage). Rats were allowed to eat a standard diet and drink ad libitum and adapted to the experimental conditions at 20 ± 2°C, humidity 60 ± 5% with a fixed 12 h artificial light period for one week.

### 2.2. Chemicals and Reagents

Trigonelline hydrochloride (trigonelline, purity 100%) was brought from Sigma-Aldrich Trading Co., Ltd, USA. Sitagliptin phosphate (sitagliptin, Januvia) was provided by Merck Pharmaceuticals, White-House Station, NJ, USA. Streptozotocin and *β*-actin polyclonal antibody were brought from Sigma Chemicals, St. Louis, MO, USA. Rabbit polyclonal antibody to GLP-1R and total and phosphorylated p38 MAPK were brought from Santa Cruz, USA. Insulin radioimmunoassay kit was provided by Beijing North Institute of Biological Technology, China. The kits of hemoglobin A_1c_ (HbA_1c_), total cholesterol, triglyceride, superoxide dismutase (SOD), malonaldehyde, and protein assay were obtained from Nanjing Jiancheng Bioengineering Institute, China. Other chemicals were of analytical reagent grade from commercial sources.

### 2.3. Induction of Diabetes and Drug Treatment

Four-treatment groups were defined: control, diabetes, diabetes plus trigonelline, and diabetes plus sitagliptin. Diabetes was induced by low-dose streptozotocin treatment (35 mg/kg, i.p.) and high-carbohydrate/high-fat diet (70% standard diet, 12% lard, 9% yolk powder, and 9% plantation white sugar) [29]. The animals with fasting blood glucose level of above 16.7 mmol/L were used as diabetic ones two weeks after streptozotocin injection. Trigonelline (40 mg/kg) and sitagliptin (4 mg/kg) were mixed daily with a vehicle consisting of the standard diet to diabetic animals for 48 weeks. The standard diet or the high-carbohydrate/high-fat diet was given only after the vehicle was completely ingested by the animals. There were 10 rats in each group. Animal weight was measured every 2 weeks throughout the experiment and the drug dose was accordingly adjusted. Fasting blood glucose levels were measured every 8 weeks to document the persistence of diabetes. After an 8 h fast, one drop of tail blood was analyzed using a standard Glucometer (OneTouch; LifeScan Inc., Milpitas, CA).

### 2.4. Cold Immersion Test and Hot Immersion Test

The animals were subjected to cold immersion test (10°C) and hot immersion test (45°C), which is the immersion of the rat tail in water maintained at the mentioned temperature and then tail flick latency or signs of struggle was observed. The cut off time was 15 s [30].

### 2.5. Nerve Conduction Studies

Nerve conduction velocity (NCV) was recorded as previously described [31, 32]. For the sciatic nerve, the recording electrodes (AD Instruments, PowerLab/16SP) were placed in the dorsum of the foot, and the stimulating electrodes at the knee and sciatic notch. For the sural nerve, the anode was placed on the third toe of the foot, and the cathode was placed on the heel of the foot. The cathode and anode were placed 5 mm apart. The frequency band was inclusive of two 10 Hz muscle potential recordings (orthodromic, motor) and 10 2 Hz potential recordings (antidromic, sensory).

### 2.6. Tissue Harvest

Forty-eight weeks after drug treatment, rats were anaesthetized by i.p. sodium pentobarbital (60 mg/kg) and opened the abdominal cavity. Cannula was inserted in portal vein and portal blood samples were collected in tubes containing dipeptidyl peptidase IV inhibitor (10 mL/mL, ADL Research, Santa Barbara, CA, USA) via the portal vein, and a blood sample (50 *μ*L) was used for measurement of HbA_1c_. Blood samples were allowed to clot to get the serum which was stored at −70°C for assessment of GLP-1 (7–36) amide and other biochemical parameters. Rats were then killed immediately and tissues were harvested as previously described [33, 34]. The left sciatic nerve was dissected and rapidly frozen by immersion in liquid nitrogen for gene and protein expression analysis. The right sciatic nerve was dissected, and parts of it were immediately submerged in ice-cold antioxidant buffer, rapidly frozen by immersion in liquid nitrogen, and stored at −70°C for quantification; other parts were fixed for transmission electron microscope.

### 2.7. Gene Expression

Total RNA from frozen tissue was extracted using the RNA-out kit. Complementary DNA was synthesized with a cDNA synthesis kit. The real-time PCR measurement of individual cDNA was performed using SYBR green dye to measure duplex DNA formation with the ABI Prism 7500 Sequence Detection System (Applied Biosystems, Foster City, CA), normalized to the amount of *β*-actin RNA, and analyzed by the 2^−∆∆CT^ method [35]. The specific PCR primers of GLP-1R were used: 5′-TGA ACC TGT TTG CAT CCT TCA-3′ and 5′-ACT TGG CAA GCC TGC ATT TGA-3′ (Accession No. NM_021332) as described [36].

### 2.8. Protein Expression by Western Blot

Western blot analysis was carried out as we previously reported [37]. For each experiment samples were run in duplicates. The densitometry analysis of the image was performed by Image-Pro Plus 6.0 (Media Cybernetics, Silver Spring, USA).

### 2.9. Plasma Biochemical Parameters Measurement

HbA_1c_, serum total cholesterol (TC), and triglyceride (TG) were measured by HITACHI 7170 automatic biochemistry analyzer (HITACHI, Japan) using commercial kit, respectively. For GLP-1 contents determination, blood was drawn into heparinized tubes containing EDTA and dipeptidyl peptidase IV inhibitor (inhibits degradation of GLP-1 by the dipeptidyl peptidase IV enzyme present in serum) by radioimmunoassay (Beijing gersion Bio-Technology Co., Ltd, China) [20]. SOD activity was assayed by the method of Kakkar et al. [38]. Malonaldehyde content was estimated by the method of Draper and Hadley [39]. Serum insulin concentration was measured by commercial kits according to the manufacturer's instructions. Insulin sensitivity index = log (1/fasting plasma glucose × serum insulin).

### 2.10. Micropathological Measurement

For transmission electron microscopy, 3-4 mm lengths of sciatic nerve of different groups of rats were obtained and fixed overnight in 2% glutaraldehyde (pH 7.3) in 0.1 mol/L phosphate buffer saline at 4°C. The specimens were then fixed in 2% osmium tetroxide (0.1 mol/L), dehydrated through a graded series of acetone, and embedded in araldite. One-micron sections were cut and then stained with toluidine blue. Suitable areas for ultrastructural study were chosen after examining 1 *μ* sections (70–80 nm), were cut on an LKB-5 ultramicrotome (Sweden) using a diamond knife and sections were mounted on a copper grid and stained with uranyl acetate and Reynolds lead citrate [40]. They were examined and photographed using a transmission electron microscope (Hitachi-7500, made by Hitachi, Japan).

### 2.11. Statistical Analysis

Data were expressed as mean ± SD. All the grouped data were statistically performed with SPSS 13.0. Significant differences between means were evaluated by one-way analysis of variance (ANOVA), followed by multiple comparisons with least significant difference (LSD) test or Tukey's test where appropriate. *P* < 0.05 was considered to indicate statistical significance.

## 3. Results

### 3.1. Effects of Drugs on Serum Glucose, Insulin, Lipid Parameters, and Body Weight

**Figure 1:**
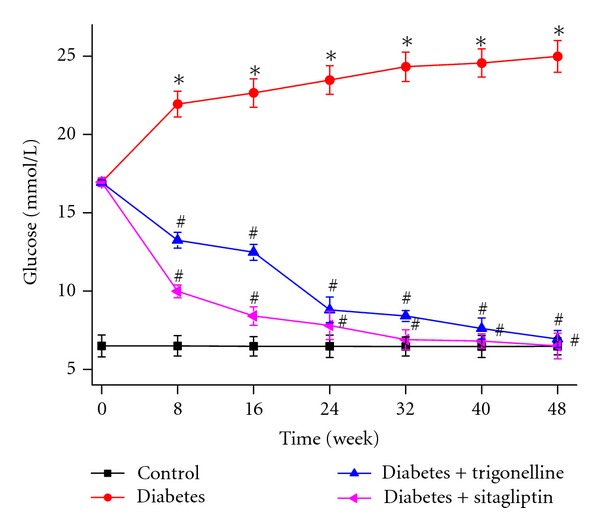
Effect of trigonelline on glucose of diabetic rats. Data are given as mean ± SD (*n* = 10). *P < 0.01 versus control rats; ^#^ *P* < 0.01 versus diabetes rats.

Before drug administration (week 0), diabetic rats had high baseline fasting blood glucose levels. Blood glucose level of diabetic rats increased significantly comparing to that of the control ones. During drug treatment, trigonelline gradually decreased fasting blood glucose to normal level (Figure 1). HbA_1c_ level of diabetic rats was significantly higher than that of the control ones. Treatment with trigonelline and sitagliptin for 48 weeks reverted the increased diabetic HbA_1c_ level to near the control ones. Diabetic rats had significant increased-serum insulin concentration and decreased-insulin sensitivity index when compared with control ones. Trigonelline treatment reversed serum insulin level and insulin sensitivity index, but did not for sitagliptin. The TC and TG levels of diabetic rats were significantly higher than those of the control ones. Treatment with trigonelline for 48 weeks significantly decreased TC and TG levels, but sitagliptin did not affect these lipid metabolic parameters. Control rats grew faster than the other group ones and body weight of diabetic rats continued to increase (data not shown). The weight gain between initial (week 0) and final (week 48) body weight of control rats were remarkably higher than that of the other group ones. At weeks 48, trigonelline, but not sitagliptin, treatment obviously reduced diabetic weight gain (Table 1).

### 3.2. Effects of Drugs on NCV and Nociception

In 48th week postdiabetes, diabetic rats showed significant decrease in both motor and sensory NCV as compared to the age-matched control rats. Treatment with trigonelline and sitagliptin for 48 weeks significantly reversed the motor and sensory nerve conduction impairment in diabetic rats (Table 2).

Tail flick latency in 48th week was significantly decreased in diabetic animals in both cold as well as hot immersion test as compared to control animals. This decrease in tail flick latency was reversed significantly in treatment with trigonelline and sitagliptin (Table 2).

### 3.3. Electron Microscopic Analysis of Sciatic Nerve

**Figure 2:**
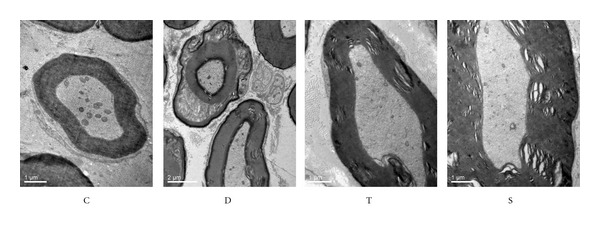
Effects of trigonelline on the micromorphology of sciatic nerve. C, control rats; D, diabetic rats; T, trigonelline-treated diabetes; S sitagliptin-treated diabetes.

Ultrastructural examination showed that sciatic nerve of control rats has normal micropathological morphology with integrity structural of nerve fibers and myelin, concentric circle-like arrangement of lamellar myelin, uniform electron density within axons, and normal structure of Schwann cells, mitochondria, and unmyelinated fibers (Figure 2(C)). But diabetic rats showed significant separation of myelinated nerve fiber myelin lamellar, lost layered-structure, disordered arrangement, faded electron density, thinner axons, neurofilament disorder, and swollen mitochondria of Schwann cells (Figure 2(D)). The protective effects of trigonelline were evident with ameliorated micropathology of diabetic sciatic nerve (Figure 2(T)), the same as sitagliptin (Figure 2(S)).

### 3.4. Effects of Drugs on GLP-1 Level in Serum and GLP-1R Expression in Sciatic Nerve

**Figure 3:**
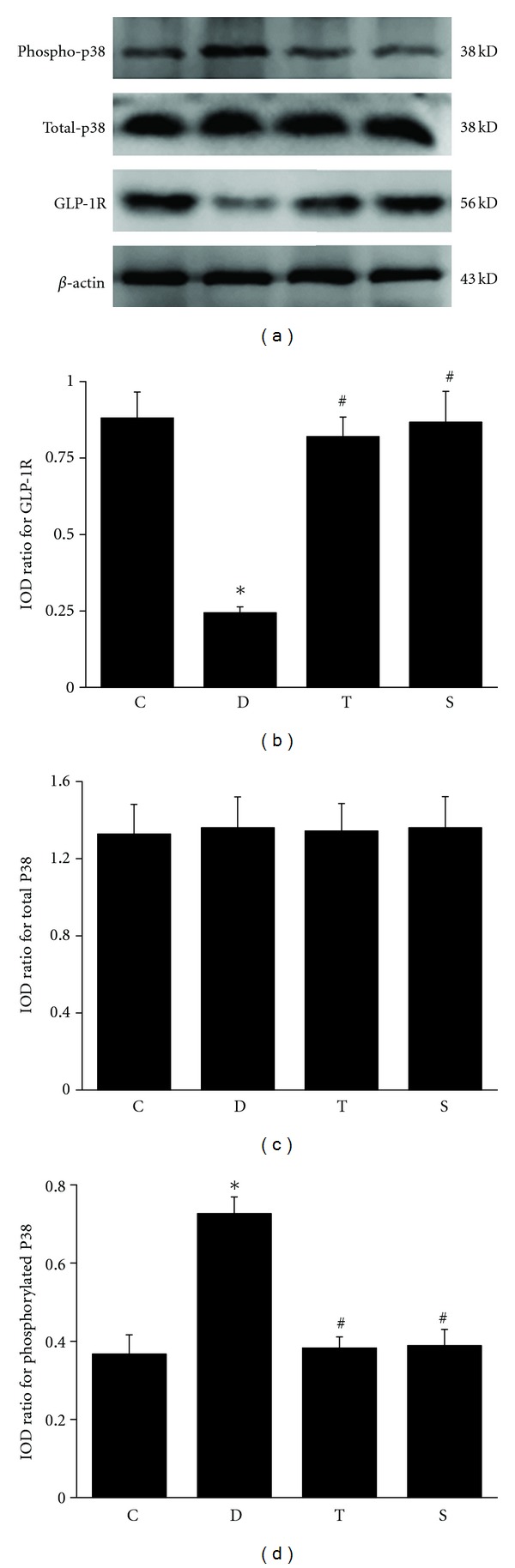
Effects of trigonelline on GLP-1R protein expression in sciatic nerve. Densitometric analysis of the bands is expressed as integrated optical density (IOD), corrected for the corresponding *β*-actin. C, control rats; D, diabetic rats; T, trigonelline-treated diabetes; S sitagliptin-treated diabetes. Data are given as mean ± SD (*n* = 10). *P < 0.01 versus control rats; ^#^ *P* < 0.01 versus diabetes rats.

GLP-1 level in serum and GLP-1R mRNA and protein expression in sciatic nerve were all significantly decreased in diabetic rats. Forty-eight-week treatment with trigonelline and sitagliptin significantly increased GLP-1 level in diabetic serum to near that of control rats. Trigonelline and sitagliptin significantly increased the downregulated GLP-1R mRNA and protein expression in diabetic sciatic nerve to near those of control rats (Table 3 and Figures 3(a) and 3(b)).

### 3.5. Effects of Drugs on Total and Phosphorylated p38 MAPK Protein Expression in Sciatic Nerve

Phosphorylated p38 MAPK protein expression in sciatic nerve significantly increased, whereas total p38 MAPK expression was unchanged in diabetic rats compared with nondiabetic control ones. Forty-eight-week treatment with trigonelline and sitagliptin significantly decreased the upregulated phosphorylated p38 MAPK protein expression in diabetic sciatic nerve to near those of control rats and still did not affect total phosphorylated p38 MAPK protein expression (Figures 3(a), 3(c), and 3(d)).

### 3.6. Effects of Drugs on Superoxide Dismutase Activity and Malonaldehyde Content in Serum

Changes in the activities of superoxide dismutase and malonaldehyde in rat serum are depicted in Table 4. Significantly decreased superoxide dismutase activity and elevated malonaldehyde content were observed in serum of diabetic rats. Significant raise in the reduced superoxide dismutase activity and significant reduction in the increased malonaldehyde content were observed in diabetic rat and reverted back to near control levels after 48-week treatment with trigonelline and sitagliptin.

## 4. Discussion

DPN is a common complication of type 1 and 2 diabetes, resulting from hyperglycemia-induced oxidative stress [41]. The current study evaluated the effects of trigonelline on the development of streptozotocin-induced DPN rats. We found that: (1) trigonelline significantly improved motor and sensory NCV and nociception; (2) trigonelline has protection on the neuron tissue (sciatic nerve); (3) trigonelline reduced oxidative stress in the sciatic nerve; and (4) GLP-1R and p38 MAPK activity were regulated by trigonelline and are likely relevant to the development of DPN.

The streptozotocin-induced diabetic rat is an established model of type 2 diabetes that develops a profound neuropathy [42]. Using a low-dose streptozotocin and a high-carbohydrate/high-fat diet protocol, we confirmed diabetes induction and animal survival for 48 weeks. The streptozotocin-induced diabetic rats developed increased fasting blood glucose, and HbA_1c_ and gained weight relative to control rats, consistent with previous reports [29]. Forty-eight-week treatment with trigonelline had anti-hyperlipidemic effects and lowered blood glucose, HbA_1c_, and body weight on DPN rats. As dyslipidemia is a significant contributor to the DPN development, hypolipidemic agent may prevent or reverse diabetes-induced nerve degeneration [43].

We demonstrated the effects of trigonelline on GLP-1R/p38 MAPK signaling pathway in streptozotocin-induced diabetic mice. Forty-eight-week treatment with trigonelline increased serum GLP-1 level and GLP-1R expression and decreased the upregulated phosphorylated p38 MAPK activity, but did not affect total p38 MAPK protein expression in the sciatic nerve. Although the precise mechanisms still unclear, the effects of trigonelline on DPN may be partly attributed to its regulation on GLP-1R/p38 MAPK signaling pathway. Exogenous GLP-1R activation significantly reduces glucose-dependent reactive oxygen species generation in hypothalamus [44]. The antioxidative effects of GLP-1R agonists, such as exendin-4, have regenerative effects on peripheral nervous systems. In the dorsal root ganglia of streptozotocin-induced diabetic rats, p38 were activated by oxidative-nitrosative stress [45].

Streptozotocin-induced diabetic rats developed persistent DPN after 48-week diabetes as measured by both increased thermal latency and decreased NCV. Thermal latency was significantly reduced by trigonelline treatment, and NCV studies demonstrated a corresponding trend toward improvement. We also observed the separation of myelinated-nerve fiber myelin lamellar, lost layered-structure, disordered arrangement, faded electron density, thinner axons, neurofilament disorder, and swollen mitochondria of Schwann cells of sciatic nerve by transmission electron microscopy. These ultrastructure changes were improved by 48-week treatment with trigonelline. Several studies showed that trigonelline could increase the excitability of dorsal root ganglion neurons [46], promote functional neurite outgrowth [47], and prevent both dendritic and axonal atrophy induced by amyloid *β*(25–35) in a dose-dependent manner [48]. The histological studies performed 50 weeks after diabetic induction suggest that the neuroprotection provided by trigonelline leads to long-lasting preservation of sciatic nerve and its function.

Trigonelline treatment had a hypoglycemic effect and partially corrected DPN. Trigonelline has an antioxidant effectiveness in cell-free systems, human colon cell lines [15], and on liposome peroxidation [16]. To determine whether the amelioration of DPN observed is due to a reduction of diabetes-induced oxidative stress, biomarkers of oxidative damage were assayed in serum. Serum SOD activity was significantly lowered, and malonaldehyde content was significantly increased in the streptozotocin-induced diabetic rats, consistent with previous work showing that diabetes increases oxidized lipids [49, 50]. Our findings are consistent with the oxidative stress model of DPN [41] because thermal latency is highly correlated with increased level of malonaldehyde and decreased level of SOD. Treatment with trigonelline reduced both biomarkers significantly, to levels near that of control animals. Because trigonelline corrected both hyperglycemia and reduced oxidative stress, we concluded that trigonelline promoted antioxidant activity in DPN. Many drugs with antioxidant function are effective in treating animal models of DPN, including the antioxidant response element activator resveratrol [50] and innate antioxidant, genistein, and baicalein edaravone [49, 51, 52].

In conclusion, this study identifies trigonelline as a multi-target drug alleviating several manifestations of DPN. These results provide the rationale for screening animal experimental studies of alkaloids, especially those with improved pharmacological profile, such as oral administration, fewer adverse effects, and acceptable pharmaceutical features, as potential therapeutics for DPN and even for other diabetic complications.

## Figures and Tables

**Table 1:** Effects of trigonelline on serum lipid parameters and body weight gain in diabetic rats.

Group	HbA1c (%)	Serum insulin (mU/L)	Insulin sensitivity index	Triglyceride (mmol/L)	Total cholesterol (mmol/L)	Weight gain (g)
Control	37.41 ± 8.53	33.24 ± 7.59	−2.31 ± 0.11	1.76 ± 0.24	0.76 ± 0.13	253.7 ± 18.0
Diabetes	59.44 ± 10.12*	47.16 ± 7.60*	−3.09 ± 0.06*	2.42 ± 0.46*	2.14 ± 0.18*	137.3 ± 7.6*
Diabetes + trigonelline	39.50 ± 10.75^##^	36.80 ± 8.13^##^	−2.38 ± 0.12^##^	1.85 ± 0.21^##^	0.79 ± 0.13^##^	103.0 ± 4.3^##^
Diabetes + sitagliptin	37.69 ± 7.81^##^	45.79 ± 8.01	−2.46 ± 0.11	2.56 ± 0.51	2.18 ± 0.24	139.2 ± 12.3^#^

Data are given as mean ± SD, *n* = 10. *P < 0.01, compared with control rats; ^#^ *P* < 0.05, ^##^ *P* < 0.01, compared with diabetes rats.

**Table 2:** Effects of trigonelline on motor and sensory nerve conduction velocity and tail flick latency in diabetic rats.

Group	Motor nerve conduction velocity (m/s)	Sensory nerve conduction velocity (m/s)	Tail flick latency in cold immersion (s)	Tail flick latency in hot immersion (s)
Control	55.34 ± 5.92	57.61 ± 6.85	14.5 ± 1.0	14.2 ± 0.9
Diabetes	35.04 ± 4.36*	40.17 ± 4.03*	5.2 ± 1.0*	6.3 ± 0.7*
Diabetes + trigonelline	49.74 ± 5.05^##^	51.76 ± 4.82^##^	12.4 ± 1.1^##^	12.0 ± 1.3^##^
Diabetes + sitagliptin	41.05 ± 5.27^#^	47.68 ± 3.38^##^	10.8 ± 0.8^##^	10.7 ± 1.0^##^

Data are given as mean ± SD,* n* = 10. *P < 0.01, compared with control rats; ^#^ *P* < 0.05, ^##^ *P* < 0.01, compared with diabetes rats.

**Table 3:** Effects of trigonelline on GLP-1 level in serum and GLP-1R mRNA expression in sciatic nerve.

Group	GLP-1 level in serum (pmol/L)	Sciatic nerve GLP-1R mRNA
Control	19.06 ± 2.65	1.00 ± 0.00
Diabetes	12.42 ± 1.11*	0.49 ± 0.08*
Diabetes + trigonelline	18.90 ± 1.25^#^	1.03 ± 0.12^#^
Diabetes + sitagliptin	18.27 ± 1.22^#^	1.08 ± 0.12^#^

ΔCT (threshold cycle) = CT (target gene) − CT (*β*-actin), ΔΔCT = ΔCT (other rats) −  ΔCT (control rats), relative fold = 2^−ΔΔCT^, and control rat is 1. Data are given as mean ± SD,* n* = 10. *P < 0.01, compared with control rats; ^#^ *P* < 0.01, compared with diabetes rats.

**Table 4:** Effects of trigonelline on SOD activity and malonaldehyde content in serum.

Group	SOD (U/mL)	Malonaldehyde (nmol/mL)
Control	124.04 ± 4.46	4.19 ± 0.78
Diabetes	83.94 ± 3.31*	6.07 ± 0.97*
Diabetes + trigonelline	115.30 ± 7.87^#^	4.57 ± 0.95^#^
Diabetes + sitagliptin	97.99 ± 4.95	5.91 ± 1.41

Data are given as mean ± SD,* n* = 10. *P < 0.01, compared with control rats; ^#^ *P* < 0.01, compared with diabetes rats.
